# TMJ Ankylosis: Multidisciplinary Approach of Treatment for Dentofacial Enhancement—A Case Report

**DOI:** 10.1155/2011/187580

**Published:** 2011-09-20

**Authors:** Pavankumar Janardan Vibhute, Nitin Bhola, Rajiv M. Borle

**Affiliations:** ^1^Department of Orthodontics, Sharad Pawar Dental College, Datta Meghe Institute of Medical Sciences (Deemed University), Sawangi (Meghe), Maharashtra Wardha 442004, India; ^2^Department of Oral and Maxillofacial Surgery, Sharad Pawar Dental College, Datta Meghe Institute of Medical Sciences (Deemed University), Sawangi (Meghe), Maharashtra Wardha 442004, India

## Abstract

This report describes the multidisciplinary phasewise treatment of a 20-year-old female patient having unilateral right TMJ bony (true) ankylosis whose mouth opening was restricted to 2 mm and mandibular retrognathism; additionally, she was also suffering from speech problems, snoring, difficulty in breathing, and low level of self-esteem and self-confidence. Bilateral gap arthroplasty and temporalis myofascial graft interpositioning through preauricular approach were done in surgical phase followed by the aggressive jaw physiotherapy in postsurgical period. Oral prophylaxis and restorations were followed by the fixed orthodontic therapy to resolve bimaxillary protrusion. Advancement sliding genioplasty was performed to enhance the chin button. Speech therapy and psychological counseling were also performed from time to time to boost up the self-esteem and self-confidence. At the end of treatment, facial esthetics was improved considerably and patient got over the impact of disfigurement, impaired functions, and psychosocial stigma. Rationale to use the multidisciplinary team approach in treatment of such cases is discussed.

## 1. Introduction

Ankylosis of temporomandibular joint (TMJ) is an intracapsular union of the disc-condyle complex to temporal articular surface that restricts mandibular movement, including the fibrous adhesions or bony fusion between condyle, disc, glenoid fossa, and articular eminence [1]. TMJ ankylosis is more commonly associated with trauma (13–100%), local or systemic infection (10–49%), or systemic diseases (100%), such as ankylosing spondylitis, rheumatoid arthritis, and psoriasis. However, it can also occur congenitally or secondary to severe rheumatoid arthritis or to tumors in the area of TMJ. Ankylosis can also occur as a result of TMJ surgery [2–5].

It is a serious and disabling condition that may cause problem in facial growth, mastication, swallowing, digestion, speech, appearance, and poor oral hygiene with rampant caries. Facial asymmetry develops if only one side is affected. Disturbances of facial and mandibular growth and acute compromise of the airway invariably result in physical and psychological disability [6–14].

Severity of ankylosis is diagnosed by evaluating the degree to which mouth opening is restricted. X-rays, CT scans, or MRI tests determine the abnormality in the bony or soft tissue formations in the joint [15]. The treatment of TMJ ankylosis poses a significant challenge because of technical difficulties and high incidence of recurrence [16]. Team approach is required for resolving functional, esthetic (cosmetic), psychological (emotional), or social problems associated with ankylosis. Report of an adult female is presented here whose treatment was carried out in a stagewise protocol.

## 2. Case Report

### 2.1. Diagnosis and Treatment Plan

**Figure 1:**
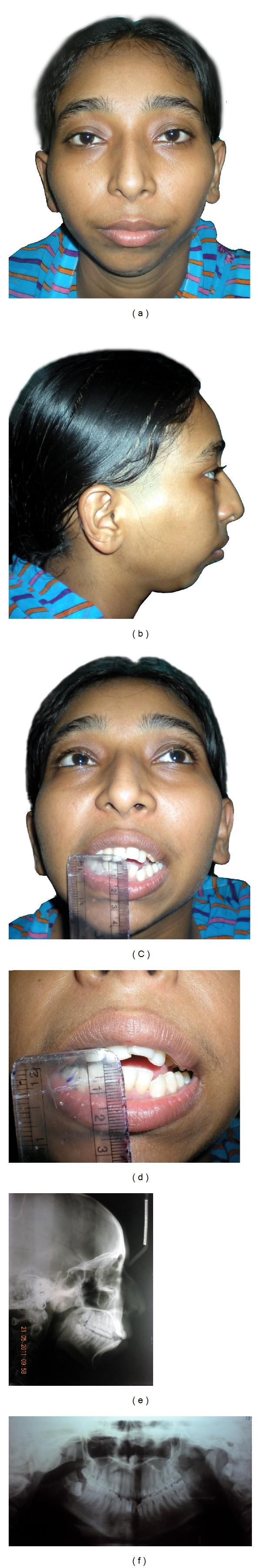
(a)–(f) pretreatment, before TMJ ankylosis surgery. Mouth opening restricted to 3 mm only.

A 20-year-old female patient reported with the chief complaint of an inability to open mouth, restricted jaw movement, and poor esthetics. She had skeletal and dental class-II malocclusion. Thorough clinical and radiographic examination revealed the case of unilateral right side bony TMJ ankylosis. On right side, ramus height and mandibular body was small, with increased gonial angle. Her feeding was characterized by an inability to masticate food, limiting intake to liquids or semisolids. She had a convex profile, severely retrognathic mandible, absence of chin button, and bimaxillary protrusion (Figure 1). Mandibular right third molar was horizontally impacted. Intraoral examination revealed mouth opening restriction of 2 mm. Upper and lower anterior teeth were malaligned and proclined due to lack of available apical basal bone. Few posterior teeth were carious and had calculus. She complained about the snoring and difficulty in sleeping on lying down. She was also the sufferer of emotional, social, and psychological disturbances with low level of self-confidence and self-esteem.

Treatment was planned in following stages:

1. surgery: gap arthroplasty and temporalis myofascial graft interpositioning through preauricular approach,
2. physiotherapy,
3. restoration of carious teeth and oral prophylactic measures to enhance hygiene,
4. fixed orthodontic mechanotherapy with all 1st premolar extractions to resolve bimaxillary protrusion,
5. advancement sliding genioplasty,
6. speech and functional therapy,
7. psychological counseling.

### 2.2. Treatment Progress

**Figure 2:**
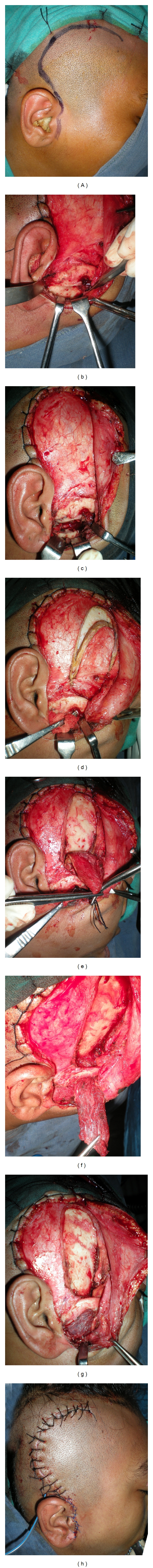
(a)–(h) gap arthroplasty and temporalis graft interpositioning through preauricular approach.

**Figure 3:**
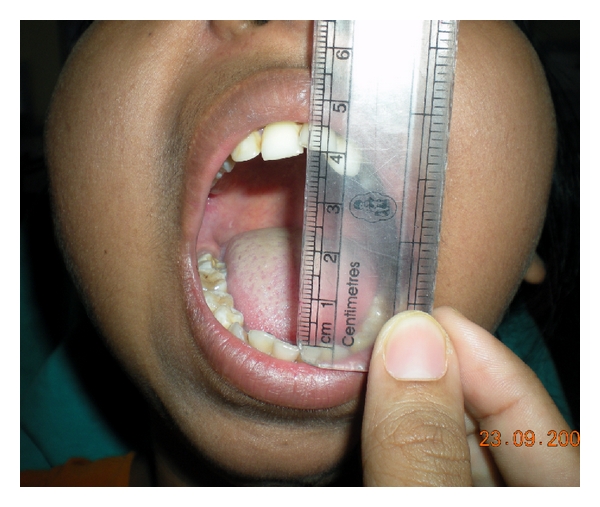
3 months after surgery mouth opening increased up to 4 cm.

**Figure 4:**
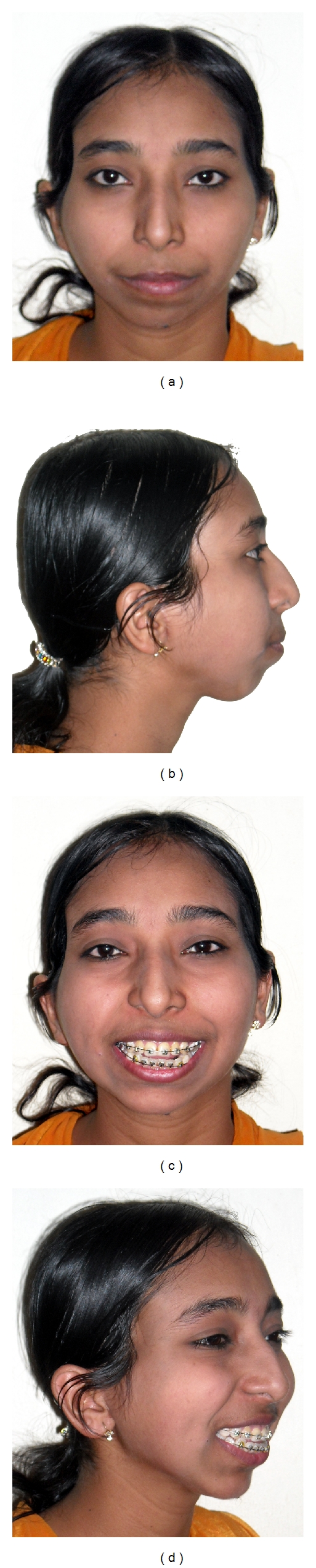
(a)–(d) facial photographs at commencing of orthodontic treatment for bimaxillary protrusion and before genioplasty.

**Figure 5:**
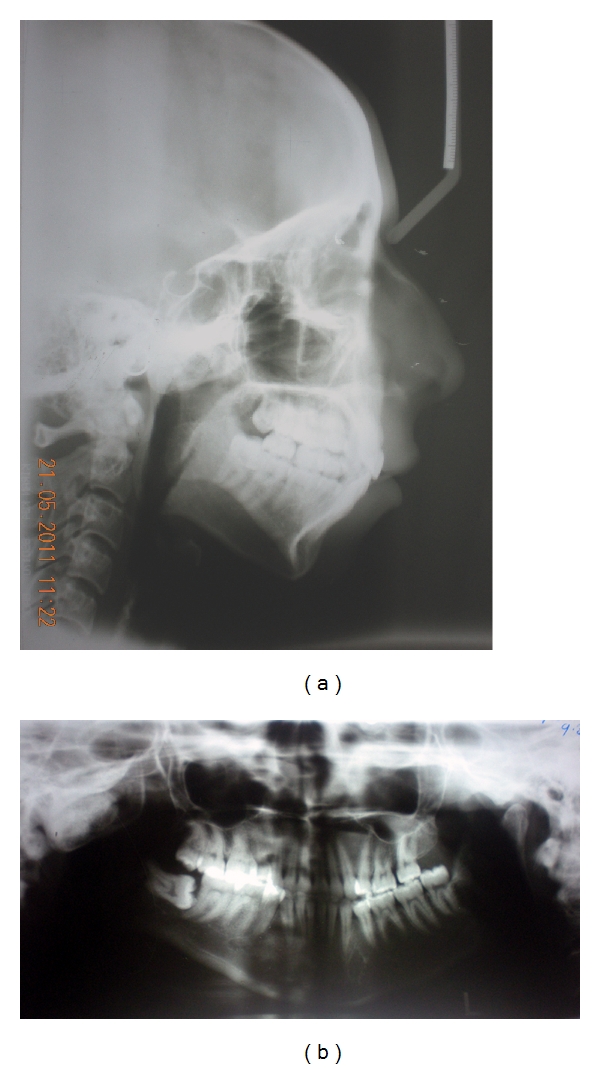
(a)-(b) radiographs after completion of orthodontic treatment and before genioplasty.

1. The initial surgery was accomplished under general anesthesia. At right TMJ, gap arthroplasty was performed through the preauricular approach (Figure 2). After exposure and identification of the site of the ankylosis, aggressive excision of the fibrous and/or bony mass was carried out with round bur and chisel until the mandibular movement was achieved. Next the glenoid fossa was recontoured as necessary (as per gap opening). For total TMJ reconstruction, after resection a costochondral graft was put in place in order to reconstruct the TMJ. Temporalis myofascial graft was interpositioned successfully [17, 18].
2. Physiotherapy: after surgery, extensive physiotherapy usually plays a crucial role in restoring normal TMJ function. Masticatory muscles, lips, and tongue exercises were proposed to increase mobility of mandible. To stimulate normal mastication, chewing on a small rubber tube was recommended. Aggressive use of continuous passive was employed, movement and tongue blades were used. Results were promising, and 4 cm range of mouth opening was achieved and satisfactorily maintained after surgery (Figure 3).
3. Restorative and oral prophylactic care: once the goal of mouth opening to its maximum by the patient was achieved, all carious teeth were restored and she was trained and learned to take the care of daily prophylactic oral hygiene measures.
4. Fixed orthodontic mechanotherapy was initiated for aligning and establishment of occlusion using fixed bonded orthodontic appliance (Figure 4). After completion of alignment and leveling in 2 and half months, all first premolars were extracted and space closure was done with maximum conservation of posterior anchorage. After 14 months of treatment, the patient showed a good class-I dental relationship, with the upper and lower anterior teeth retracted and uprighted into near-normal positions over the basal bone. Space closure was completed without the development of an anterior open bite or deep overbite. With the retraction of the lips, the patient's profile and smile improved (Figure 5). After debonding of fixed appliance, upper wraparound and lower Hawley's removable retainers were delivered.
5. Genioplasty: 6 months after completion of orthodontic treatment, sliding advancement genioplasty was performed. Chin advanced 8 mm, and fixation was performed with titanium plate.
6. Speech and functional therapy: concerns regarding speech were thought to be equally significant with appearance in contributing to low self-esteem in this patient [19]. Unusual speech due to jaw thrusting was the problem faced by the patient. She was thoroughly evaluated for the speech therapy. Problem observed in patient was mixed type of articulation disorder included omission, substitution, and distortion types. Patient was prepared for the speech therapy right from initial visit in clinic, and major training was started after possible sufficient jaw movements after TMJ surgery. Articulation defects were well improved after joint surgery and orthodontic dentoalveolar corrections. Lisping of sound was eliminated completely after orthodontic corrections.
7. Psychological counseling: psychometric tests can identify the adversity in the experience of TMJ ankylosis having facial poor appearance. It was attempted to evaluate the thoughts and feeling of a disfigurement in face in pretreatment and posttreatment stages using standardized psychometric questionnaires that have been developed, validated, and used by social scientist and psychologist.

## 3. Result

**Figure 6:**
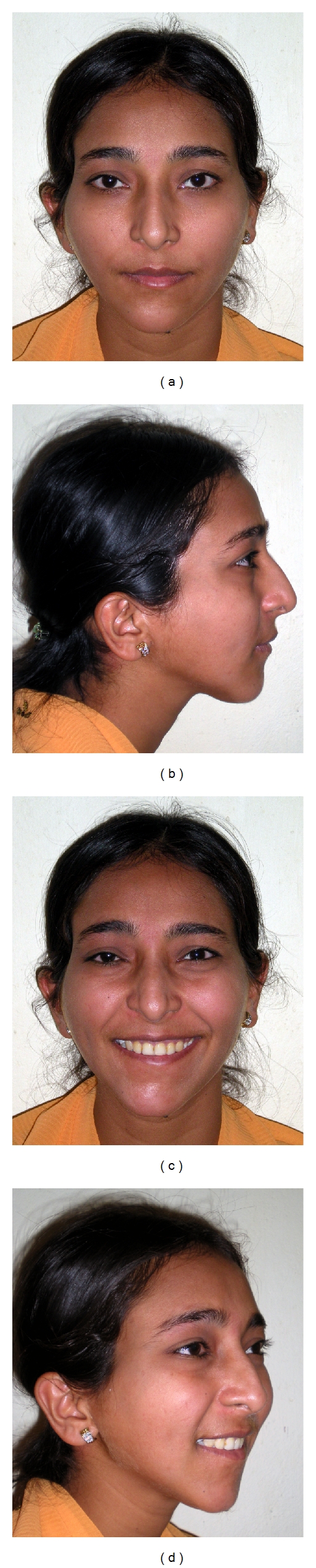
(a)–(d): after completion of orthodontic treatment and 7 mm advancement genioplasty, facial esthetics was improved considerably.

**Figure 7:**
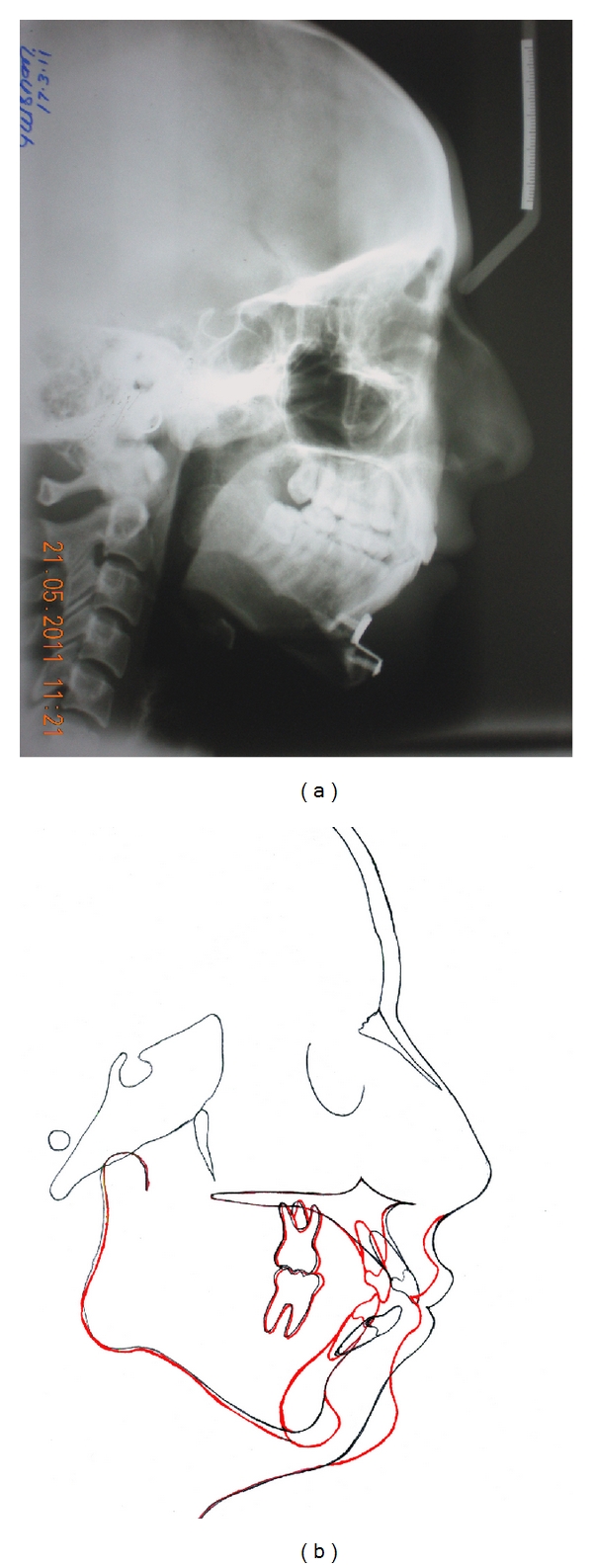
(a) Postgenioplasty cephalogram, (b) pre-and posttreatment superimposition.

Profile of patient was improved from retrognathic to orthognathic, and mouth opening was increased up to 4 cm (Figures 6 and 7). The patient showed favorable results not only in terms of esthetics and function but also profoundly positive influence on the psychological development, self-esteem, and self-confidence. Parents and the patient agree on the satisfaction with clarity and fluency of speech.

## 4. Discussion

A case is considered to represent adolescents who present unique psychosocial adjustment concerns with craniofacial anomaly. TMJ ankylosis impacts facial and head features differently, resulting in differences in both appearances and speech. Management of the psychosocial adjustment of patient with poor facial appearances has moved from development of adaptation towards the optimum results deserved by the patient from the possible team approach [20–28]. In the case presented here, there was significant improvement obtained by employing treatment through phasewise multidisciplinary approach.

This patient report demonstrates that the well-planned stagewise treatment by various specialists serves the best possible approach ever deserved by the patient to get over the impact of disfigurement, impaired functions and psychosocial stigma. Specialists involved in team care were orthodontist, oral and maxillofacial surgeon, physiotherapist, speech therapist, psychiatrist, endodontist, and periodontist. Biggest advantage of multidisciplinary team care was achieving the goal of functional efficiency, structural stability,vesthetic harmony, and psychosocial competency.

## 5. Conclusion

Multidisciplinary approach by team work for treatment of TMJ ankylosis serves towards a better outcome in order to capture the richness of experience in the lives of patient and boosting the level of confidence along with normal form, function, and stability.
